# Application of P(VDF-TrFE) Glass Coating for Robust Harmonic Nanoparticles Characterization

**DOI:** 10.3390/mi12010041

**Published:** 2021-01-01

**Authors:** Svitlana G. Ilchenko, Volodymyr V. Multian, Ruslan A. Lymarenko, Victor B. Taranenko, Salvatore A. Pullano, Antonino S. Fiorillo, Volodymyr Ya. Gayvoronsky

**Affiliations:** 1 International Center “Institute of Applied Optics”, National Academy of Science of Ukraine, Kudryavska Street 10G, 04053 Kyiv, Ukraine; ilchenko@iao.kiev.ua (S.G.I.); kit@iao.kiev.ua (R.A.L.); victor.taranenko@iao.kiev.ua (V.B.T.); 2 Institute of Physics, National Academy of Science of Ukraine, Prospect Nauky 46, 03028 Kyiv, Ukraine; multian.v.v@gmail.com (V.V.M.); vlad@iop.kiev.ua (V.Y.G.); 3 Department of Health Sciences, University “Magna Græcia” of Catanzaro, 88100 Catanzaro, Italy; nino@unicz.it

**Keywords:** P(VDF-TrFE) coating, laser beam self-action, optical damage threshold, third harmonic generation, harmonic nanoparticles

## Abstract

Polyvinylidene fluoride and its copolymers are a well-known family of low-cost ferroelectric materials widely used for the fabrication of devices for a wide range of applications. A biocompatibility, high optical quality, chemical and mechanical durability of poly(vinylidene fluoride–trifluoroethylene), (P(VDF–TrFE)), makes it particularly attractive for designing of effective coating layers for different diagnostic techniques. In the present work, the nonlinear optical characterization of P(VDF-TrFE)-coating films deposited onto a glass substrate was done. Advantages of the coating application for cells/substrates in the field of multiphoton imaging the efficiency of such coating layer for long-duration characterization of so-called harmonic nanoparticles (HNPs) were shown. The influence of glass surface protection by P(VDF-TrFE) film from an effect of HNPs sticking to the walls of the flow-cell was analyzed for effective studying of the optical harmonics generation efficiency of HNPs making the analysis more robust.

## 1. Introduction

The main idea of the work is to study the perspective of using new type of cuvette coating for application in bioimaging systems and different nanoparticles characterization techniques. We present results with coating based on poly(vinylidenefluoride-trifluoroethylene) P(VDF-TrFE) copolymer with chemical formula (CH_2_-CFH)_n_. In general, it is optically transparent, semi-crystalline piezoelectric polymer, where chains exhibit strong preferential orientation [1,2,3,4]. This lightweight, low cost, ultra-sensitive, and high deformable polymer is mainly used in areas requiring excellent chemical resistance, high purity, and excellent mechanical properties [5]. It is also used in piezoelectric [6] and electrostriction applications [7] as wearable electronics [8], flexible tactile sensing devices [9,10,11], ultrasonic measurement devices [12,13,14], human skin, and many others [9,15,16,17,18,19,20]. Because of an efficient nonlinear optical response, P(VDF-TrFE) copolymer is promising for many optical applications, such as sensors [21,22], ultrafast switches, ultrashort pulsed lasers, and many others. In addition, we should mention the utilization of the PVDF-based composites as they superhydrophobic and antibacterial material [23,24] that make them perspective for designing of effective coating layers.

For practical applications in the field of bioimaging, the efficiency of the optical harmonics generation of harmonic nanoparticles (HNPs) should be well studied. In this case, diffrent techniques should be applied for the characterization of HNPs. Among them are the hyper-Rayleigh scattering technique [25,26] and the interface scanning technique [25], which can be applied for studying the second harmonic (SH) and third harmonic (TH) generation correspondingly. In case of hyper-Rayleigh scattering technique, the SH signal can be easily readout from the center of the cuvette with colloidal suspension of NPs.

The interface scanning technique can be used to readout the TH signals generated from the interfaces between two media inside of the cuvette-colloidal suspension and glass. Comparison of the TH signal at the interface colloid/glass and glass/air allows estimating the efficiency of TH generation of suspension and extracting the efficiency of HNPs by applying the effective media model. This technique was first designed for the characterization of liquids and gases and then it was optimized in order to study the different objects, such as different nanoparticles [26], red blood cells [27,28], embryonic development [29], organic solvents [30], neurons [31], and skin biopsy samples [32].

However, the high sensitivity of the interface scanning technique significantly depends on the quality of the interface surface. Considering the small readout volume, it is important to minimize the effects of hydrothermodynamical motion of HNPs (or other types of studied objects) in the area close to the waist of the focused laser beam, sticking to the surface of the cuvette, and agglomeration and ablation effects [25]. As a result, each measurement should be done fast and the cuvette must be cleaned each time, which can be difficult on practice. An alternative solution for such problem is based on using the system with a flow cell that provides effective refreshing colloidal suspension of studied material in readout volume per each laser shot [25] for correct response averaging with minimal fluctuation of the signal. Such approach can significantly increase the efficiency of measurements, but it cannot completely solve the problems with sticking and requires periodical cleaning of the cell walls.

In order to solve the problem with HNPs sticking to the cuvette’s surface, a specific coating can be used. The main requirements to this coating are high durability (optical and chemical), low absorption, and small impact to the resulting TH signal at the coated interface in comparison with the uncoated one.

We used zinc oxide (ZnO) harmonic nanoparticles (HNPs) to investigate the interaction with polymer interface. ZnO is a widely used material in optics, optoelectronics, biosensorics research because of its non-toxicity, biocompatibility, and easy of fabrication. The composites with ZnO nanoparticles (NPs) demonstrated both corrosion protective effect [24] and UV-induced robust self-cleaning ability [23]. Moreover, the influence of ZnO inhalation on the airway inflammatory markers [32], ZnO nanoparticles as metal-based drugs [33,34], and functionalized ZnO used to treat cancer cells were studied [35]. HNPs are a new type of nonlinear optical (NLO) markers for biological systems based on inorganic oxide nanocrystals with a noncentrosymmetric lattice that effectively converts a frequency of laser radiation [36,37,38]. This term was introduced for the designation of a new broad class of NPs that can simultaneously generate SH, TH, and higher optical harmonics with high efficiency. HNPs markers are promising for applications in the field of bioimaging, because of the tunable bands position of harmonic signals, the high depth of imaging [35], and photostability for long-duration observation.

The obtained result presented in this work can be important for a more precise scientific tool—a third harmonic generation microscopy that is a flavor of multiphoton microscopy which can provide images of biological samples based on spatial variations in third-order nonlinear susceptibility (χ^(3)^), refractive index (n), and dispersion (n_3ω_–n_ω_) [29,39]. This method is widely used to obtain structural information about a variety of biological specimens. Its nature allows depth-resolved imaging [40] of inhomogeneities, with practically no background from surrounding homogeneous media. With an appropriate illumination geometry, third harmonic generation microscopy is shown to be particularly suitable for imaging of biogenic components and is perspective for studying of biological tissues [29,41]. Applying of P(VDF-TrFE) coating with well-characterized NLO properties for substrates in Third Harmonic Generation (THG) microscopy can significantly increase the precision of this approach.

## 2. Materials and Methods

### 2.1. Experimental Setup

**Figure 1:**
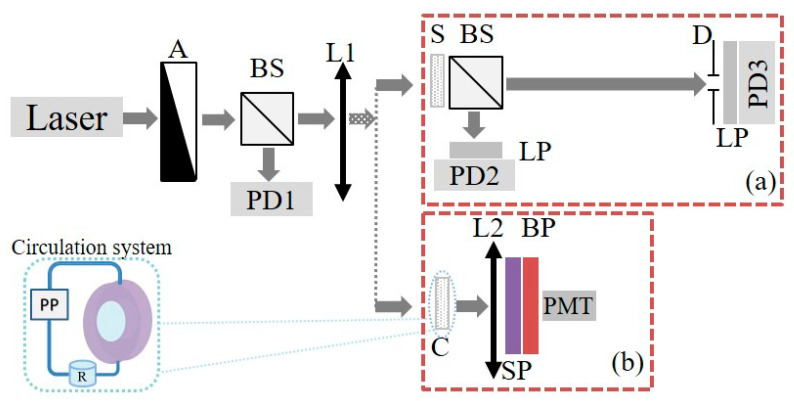
(**a**) Scheme of experimental setup for analysis of photoinduced variations of total and on-axis transmittance due to the self-action of the laser beam under ps pulsed excitation at 1064 nm; (**b**) experimental setup for interface scanning technique at TH. A—neutral density gradient filter; BS—beam splitter; L1, L2—focusing lenses; D—finite diaphragm (d= 2 mm); PD1, PD2, PD3—photodiodes; PMT—photomultiplier tube; LP—longpass filter; SP—shortpass filter; BP—bandpass filter; S—sample; C—flow cell; R—main reservoir; PP—peristaltic pump.

Designing of novel coatings for nonlinear optical applications requires precise characterization for working excitation intensity range. For this purpose a laser beam self-action analysis technique [42,43,44,45] was used (see Figure 1a). For measurements, Nd:YAG-based laser (1064 nm, FWHM 42 ps, repetition rate 40 Hz, Institute of Physics, National Academy of Science of Ukraine, Kyiv, Ukraine) was applied. Studied sample S was positioned after the waist of the focusing lens L1. Input power was varied by neutral density gradient filter A and monitored by calibrated photodiode PD1. Analysis of the total and on-axis transmittance were done by photodiodes PD2 and PD3, with longpass filters to remove the signals of optical harmonics and PL.

For measurement of the THG efficiency (see Figure 1b) the sample was positioned on the 3-axis translation stage. For the interface scanning technique, the studied sample was scanned by the waist of the focused laser beam. Generated TH signal in the forward Z-direction was collected by lens L2 and measured with a photomultiplier PMT (Hamamatsu H10721-210) placed after a shortpass and a bandpass filter to remove the pump beam and to extract the TH response at 355 nm.

### 2.2. Samples Preparation

Studied P(VDF-TrFE) copolymer films with different thickness were deposited on cover glass (see Table 1). Pellet forms of P(VDF-TrFE) were preferably dissolved in methyl ethyl ketone (MEK) with a molar ratio of 70/30. The solution was then spun onto glass coverslip at about 3000 rpm/min for 60 s. The rotational speed mainly affected the polymer film thickness after evaporation of solvent. Using a rotation speed ranging from 3000 up to 5000 rpm/min for about 60 s, a thickness in the range 1–2 µm was obtained. A calibrated process allows a control on the thickness in the nm range, while the roughness depends also on the roughness of the substrate. Poling procedure of the samples was accomplished with a constant electric field of about 2.5 MV/m per 30 min and then annealed on a hot plate at 80° C for about 30 min, in order to allow the solvent evaporation. After removing the electric field, a cooling process was carried out at room temperature. In order to analyze the influence of poling and thickness two additional samples were prepared: (i) a thin (<2 µm) unpoled film and (ii) a thick (~40 µm) unpoled film of PVDF.

To study the influence of HNPs sticking on the THG response of the interface colloid/glass the two cases were studied: (i) HNPs deposited on the surface of the substrate and (ii) colloidal suspension circulation in flow cell.

**Figure 2:**
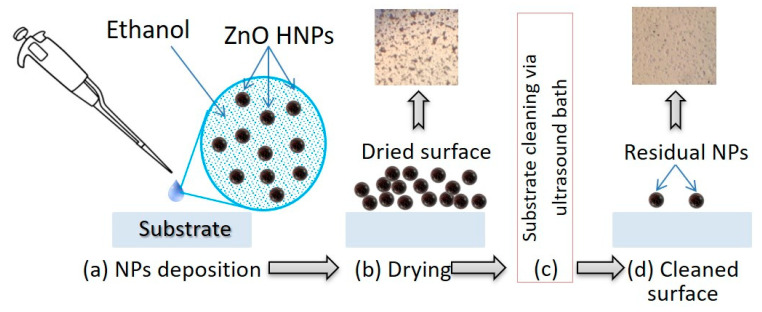
Main stages of the HNPs deposition and cleaning procedure: (**a**), (**b**) Deposition of HNPs by evaporation from colloidal suspension; (**c**) cleaning of the surface of the sample by 3 min treatment in ultrasonic bath; (**d**) drying of the “cleaned” area. Inserted photos show the dense layer of the ZnO NPs aggregates before and after cleaning in ultrasonic bath.

For analysis of the HNPs sticking to the surface of the glass, ZnO HNPs (NanoAmor, Katy, TX, USA) [46] (average diameter of NPs~150 nm) were used because of the high efficiency of SH [47], TH [25], and PL signals. Scheme of the sample’s preparation with deposited ZnO HNPs is presented in Figure 2. At the first stage an initial ethanol-based colloidal suspension (6 mg/mL) was deposited on the cover glass by a micropipette. Drying of the sample allows to obtain a homogeneous layer of NPs on the surface of the glass. This step can be interpreted as a result of longtime measurements with sedimentation/sticking of NPs during experiment with colloidal suspensions.

The typical and easiest approach (but not the most efficient, as it will be presented below) to remove HNPs from the surface is to use standard laboratory ultrasonic bath. For his step, the part of the samples covered by HNPs was inserted into the ultrasound bath filled by ethanol for 3 min and after that it was dried. The same procedure of samples preparation was realized for cover glass coated by P(VDF-TrFE). In general, the results obtained for ZnO HNPs in future can be used to develop practical solutions in case of colloidal suspensions of other types of NPs, biological and organic components.

## 3. Results and Discussion

### 3.1. Self-Action Analysis

**Figure 3:**
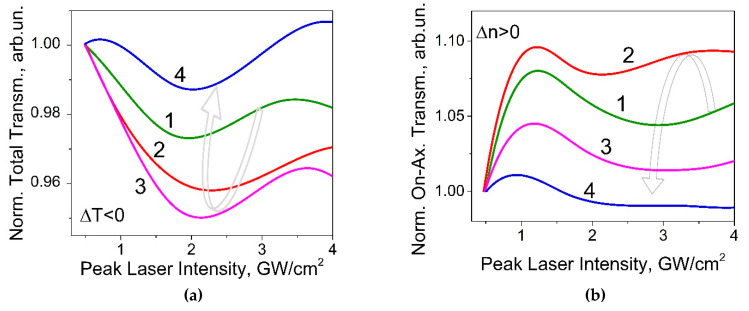
The photoinduced variations of the normalized total (**a**) and on-axis (**b**) transmittances versus the peak laser intensity of picosecond laser pulses at 1064 nm for the samples of P(VDF-TrFE) copolymer with different thickness: (1)—1 μm, (2)—1.2 μm; (3)—1.3 μm; (4)—1.5 μm.

Typically studying [25] the TH generation efficiency via the interface scanning technique was realized at intensities about 1-10 GW/cm^2^. At these levels of excitation intensity, the influence of NLO effects based on self-action of laser beam cannot be neglected. In order to analyze NLO properties of polymer, the technique based on spatial profile analysis via laser beam self-action [44] was used. Figure 3 shows the photoinduced variations of total and on-axis transmittance versus the peak laser intensity for the studied P(VDF-TrFE) samples with different thickness—one of the significant parameters for coating. It should be noted that the total (Figure 3a) and on-axis transmittance (Figure 3b) of the sample were normalized by cover glass response as apparatus function for this experiment.

Analysis of the absorptive NLO responses (Figure 3a) have shown the photodarkening effect manifestation to be less than 4% that saturates at intensities *I* > 2 GW/cm^2^ for all of the samples. At the same time the studied samples demonstrate the self-focusing effect with efficiency Re(*χ*^(3)^)~10^−7^esu (see Table 1) in the range *I* < 1 GW/cm^2^ (see Figure 3a), that corresponds to the positive photoinduced variations of the refractive index Δ*n* > 0. It saturates at intensities of about 1GW/cm^2^ and turns to self-defocusing.

**Figure 4:**
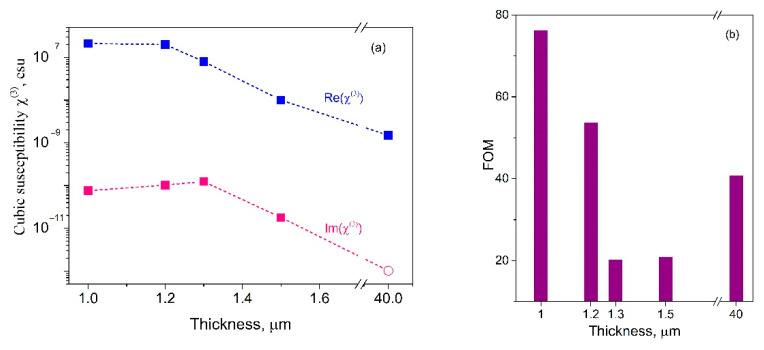
(**a**) Comparison of real Re(χ^(3)^) and imaginary Im(χ^(3)^) parts of cubic NLO susceptibility vs. thickness for coating films under picosecond laser excitation at 1064 nm. The red circle point corresponds to the absolute values of Im(χ^(3)^) for the sample №5 (see Table 1); (**b**) Variation of FOM vs. samples thickness.

Figure 4 shows the influence of the thickness of polymer layer on the nonlinear optical response. In general, the real part of cubic NLO susceptibility Re(*χ*^(3)^) is about 3 orders of magnitude higher than the imaginary part Im(*χ*^(3)^), that shows a high optical quality of the studied materials. Both Re(*χ*^(3)^) and Im(*χ*^(3)^) decrease with the increase in thickness. However, the dynamics of this variation is different. For this case, it is efficient to estimate FOM = Δ*n*/(*λ*Δα)−igure of merit, where α is the absorption coefficient (see Figure 4b). In general, it shows the ratio between the efficiency of refractive and absorptive responses of the studied material.

In case of performing measurements in a wide range of intensities, the nonlinear-optical properties of the materials are important, because they can lead to a distortion of the laser beam and a misinterpretation of the results. Analysis of FOM variation vs. samples thickness (see Figure 4b) shows that the poled films with thicknesses 1.3/1.5 μm demonstrated the low FOM = 18/19 factor (see Table 1) that reflects a reduction of the NLO refractive contribution in total optical response of the material. Collation of total/on-axis transmittances of the samples 3 and 4 have shown exact drop of the photodarkening/self-focusing effects manifestation—photoinduced variation of the mentioned transmittance at the level about 1%—for the film № 4 with thickness 1.5 μm. Increase of the film thickness of 15% vs. the film № 3 decreases at about of order of magnitude efficiencies both of refractive and of absorptive cubic NLO responses. On practice, it means that applying of this sample as coating will not significantly distort and absorb laser beam at different excitation intensities.

**Figure 5:**
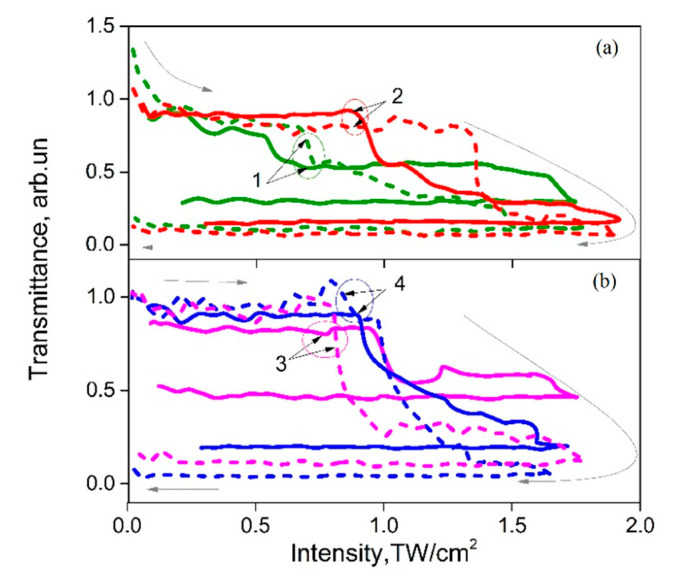
The photoinduced variations of total (solid line) and on-axis (dashed) transmittances versus the peak laser intensity at 1064 nm for the samples of P(VDF-TrFE) copolymer with different thicknesses: (1) 1.0 μm, (2)—1.2 μm, (3)—1.3 μm, (4)—1.5 μm. (**a**) Comparison of unpoled (1) and poled (2) samples. (**b**) Comparison of samples with different thickness. Arrows indicate the influence of the increase intensity up to threshold and consequent decrease.

To study the durability of coating films the photoinduced variations of the total and on-axis transmittances were analyzed in range up to 2 TW/cm^2^ (see Figure 5). We have observed significant irreversible reduction of the films total transmittances at peak laser intensities at about 1 TW/cm^2^ attributed to the optical damage threshold. Below the threshold the nonlinear optical variations of the refractive index and optical absorption were reversible along the rise and consequent reduction of the laser intensity. This fact demonstrates the high durability of P(VDF-TrFE) coating for the high-intensity excitation regimes. It should be noted that unpoled film №1 demonstrates lower threshold magnitude—about 0.7 TW/cm^2^—in comparison with the poled ones of similar thickness.

Analysis of the total and the on-axis transmittances variations (solid and dotted curves at Figure 5) demonstrates different threshold intensities that can be explained by the reorientation of the polymer molecules (poled or randomly oriented) under high excitation intensities, as it was shown for systems of liquid crystals with different thickness in comparison to the effective interaction length [48].

We compared the obtained refractive cubic NLO response efficiency Re (*χ*^(3)^) with the available reference data for PVDF-based materials (see Table 2). Most of the previous measurements were realized at wavelength 532 nm with doubled energy quanta versus our case. It was shown that at peak laser intensity 1 GW/cm^2^ the PVDF-based composites demonstrated self-focusing/defocusing effects with efficiency |Re (*χ*^(3)^)| ≤ 2.2 × 10^−8^ esu [48]. For the similar ZnO dopant type [49] the efficiency of the NLO response reduces about three times at higher excitation intensity with the decrease of dopant fraction. We have also observed a saturation of the self-focusing effect manifestation accompanied with 1-2 orders of magnitude of Re(*χ*^(3)^) drop in the studied films. In PVDF/RGO composites authors obtained pronounced optical limiting effect with suppressed NLO refractive response one [50].

In general in our work the obtained magnitudes |Re (*χ*^(3)^)|~10^−8^ esu of self-action effect manifestation at wavelength 1064 nm corresponded to the reference data, where the sign and the magnitude of Re(*χ*^(3)^) depended on the dopant type and its concentration. The opposite signs of the refractive NLO response can be attributed to the difference in excitation regimes concerning (i) laser wavelength [53], (ii) pulsed (ns vs. ps) or CW mode [54], and (iii) range of the applied peak laser intensities [44].

### 3.2. Analysis of Harmonic Nanoparticles (HNPs) Sticking Effect on Third Harmonic Generation (THG) Response of the Interface

Because of the presence of two media with different refractive index and/or cubic NLO susceptibilities, THG is observed at the material’s interfaces for tightly focused laser beam. In the case of several interfaces, for example, glass/film/HNPs, resulting signal depends on interrelation of their efficiencies [25], third-order susceptibilities *χ*^(3)^. It is necessary to know, the properties of each layer at the interface. In the case of several interfaces, it is necessary to know, the properties of each layer at the interface, for example, glass/film/HNPs-resulting signal depends on the interrelation of their efficiencies, third-order susceptibilities *χ*^(3)^.

The scanning was performed by linear translation in perpendicular direction to the fringes of different sample areas: (i) initial glass, (ii) deposited ZnO HNPs, and (iii) area cleaned by ultrasonic bath. The same was done for the sample with polymer coating.

**Figure 6:**
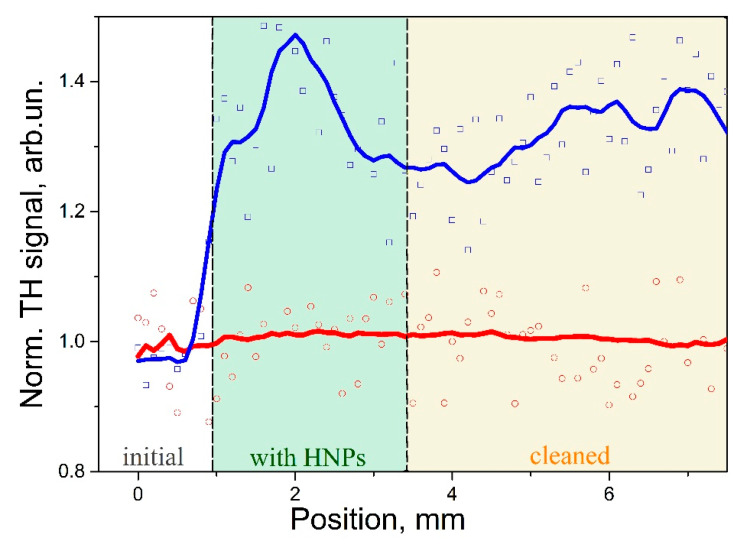
The THG signal scanning in plane of the sample under picosecond range pulsed laser excitation at 1064 nm for glass cover slip (blue) and cover glass coated by P(VDF-TrFE) (red). Filled areas correspond to the initial area, area with deposited ZnO HNPs and cleaned part in ultrasonic bath. Signals for coated and uncoated areas are normalized on the response at initial area. Solid lines obtained by moving average filtering of raw data, for better visualization of signal variation.

Obtained results of surface scanning are presented in Figure 6. In case of uncoated glass, the TH scanning signal profile can be explained by variation of ZnO HNPs concentration from initial area without HNPs through deposited layer of ZnO HNPs to area cleaned by ultrasonic bath. Obtained results demonstrate the low efficiency of cleaning in ultrasonic bath for uncoated glass without additional mechanical treatment in the cleaning process.

In contrary to the uncoated substrate, P(VDF-TrFE), coating demonstrates minimal variations of TH signal in all of the studied areas. It should be noted that the studied samples were positioned vertically during measurements, tested excitation intensities were below 500 GW/cm^2^, and examination by optical microscopy did not reveal the areas with optical damage of the coating. Taking into consideration these facts, it is possible to assume that at excitation intensities of about 500 GW/cm^2^ the HNPs can be removed from the coated surface by laser beam. However, this fact requires further detailed analysis.

### 3.3. HNPs Sticking Effect in Flow Cell

**Figure 7:**
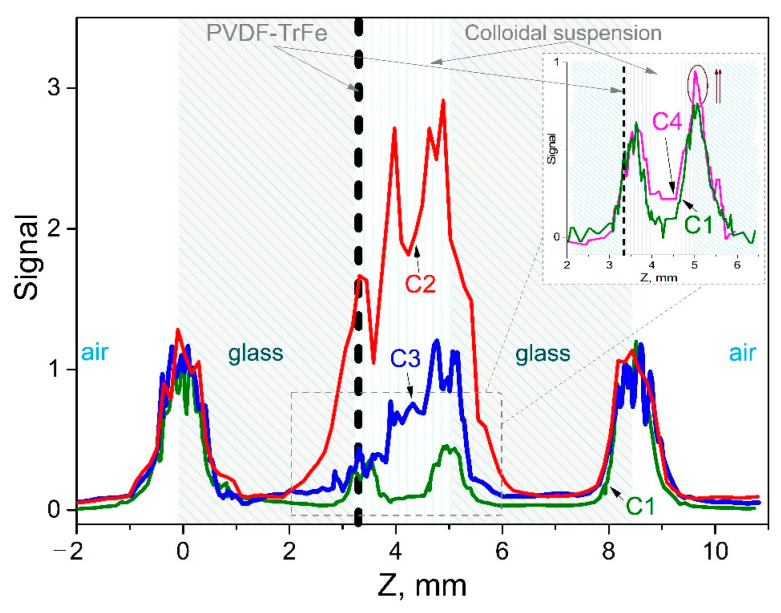
Experimental data of the TH signal measured by scanning the interface of the flow cell with different concentrations (see Table 1). C1—ethanol, C2—ZnO HNPs in ethanol (~1 mg/mL), C3—ethanol in cell after ~30 min of measurements with ZnO HNPs colloidal suspension. The insert shows the comparison of the initial ethanol response C1 and C4—ethanol after 30 min of measurements with lower concentration (~0.1 mg/mL) of ZnO HNPs colloidal suspension.

Next stage, after the study of HNPs deposited on substrate, was to provide measurements with flow-cell system and colloidal suspension circulation. Figure 7 shows the TH signal measured by scanning the interfaces of the flow cell. Experimental data represent four THG peaks, that correspond to interfaces of the cell: air/glass, glass/liquid, liquid/glass, and glass/air interfaces, respectively.

First, THG measurements at the interfaces of flow cell with circulation of ethanol were done. Comparison of the THG peaks at the coated and uncoated interfaces inside of the cell showed slight difference, due to the impact of the PVDF-TrFE layer, that should be taken into account in case of precise calibration of experimental setup.

In previous works [25,55] the typical measurements with reproducible response for low concentrations (up to 0.1 mg/mL) of colloidal suspensions were provided for a time lapse below 15 min. In order to demonstrate the efficiency of coating, in this work the concentration of HNPs was increased by ten times and the circulation time was extended to 30 min. We should mention that the experiment with high concentration of NPs was realized only to demonstrate the efficient manifestation of the HNPs sticking, being improper for the distinct measurements within the interface scanning technique.

At the next stage, the colloidal suspension was pumped up from the circulation system and replaced by clean ethanol. Obtained results demonstrate the reproducibility of the measurements at the interfaces with uncoated and coated internal walls of the cell: amplitude of the TH peak at uncoated interface increased two times while the signal at the coated interface stayed unchanged.

In results, the surface coating was not only protected from impurity, but also allowed self-cleaning under the influence of high intensity of the focused laser beam. Because of this effect and its mechanical, electrical, thermal, and chemical resistance, the polymer can be used as an effective coating layer for the substrates/cuvettes/cells interfaces to promote NLO characterization of NPs and/or biological samples.

## 4. Conclusions

An applicability of the P(VDF-TrFE) coating at the glass substrate as a part of experimental cell was studied for the third harmonic generation effect in colloids of ZnO HNPs. Conventional glass walls of the cuvette or flow cell demonstrates effect of sticking of HNPs during longtime nonlinear optical measurements that can deteriorate estimation of magnitudes of laser frequency conversion efficiency. Comparison of the flow cell windows coated with the P(VDF-TrFE) internal coating vs. the uncoated ones has shown that the coating utilization reduced HNPs sticking on its interface, being monitored by the TH signal registration. Obtained results demonstrate the reproducibility of the measurements at the interfaces with uncoated and coated internal walls of the cell: amplitude of the TH peak at uncoated interface increased two times while the signal at the coated interface stayed unchanged. As a result, such an approach can significantly increase the efficiency of long-duration measurements.

For the estimation of adhesion level, it is possible to use a polymer for the creation of spatially periodic structures (patterning). In this configuration, the response from the polymer-coated surface and the substrate can be compared. We have studied self-action effects manifestation of picosecond range pulsed laser radiation at wavelength 1064 nm in broad range of peak laser intensity up to 2 TW/cm^2^. The optical damage threshold was observed at about 1 TW/cm^2^: (i) Photoinduced variations of the refractive index and optical absorption were reversible along the rise and consequent reduction of the peak intensity below the threshold; (ii) irreversible significant reduction of the transparency was observed for the excitation above the threshold. Its magnitude depends on the coating thickness; unpoled films have lower threshold magnitude vs. the similar poled ones.

Below the optical damage threshold the P(VDF-TrFE) films demonstrated efficient refractive NLO response—self-focusing effect—with Re(*χ*^(3^)~10^−8^ esu that saturated at about 1 GW/cm^2^ excitation intensity. It accompanied with slight photodarkening of the coatings with Re(*χ*^(3^)~10^−11^ esu that corresponded to high FOM~20–80. The measurements have shown that the poled film with thickness about 1.5 μm demonstrated the FOM = 19 factor with minimal refractive and absorptive NLO responses efficiencies among the poled films. We suggest that the mentioned coating is going to be an optimal one for the HNPs diagnostics application, because of the reduction of the photoinduced refractive NLO response contribution impact on the optical harmonics generation effects studied in the cell with the interface scanning technique.

Taking into account the high optical damage threshold and laser damage resistance, high mechanical, electrical, thermal, and chemical resistance, it is possible to conclude that P(VDF-TrFE)-based coating is promising for improving the existing and designing new experimental techniques for harmonic nanoparticles characterization. The obtained results are promising for utilization in multiphoton microscopy diagnostics both of nanoparticles and of biological objects.

## Figures and Tables

**Table 1:** The real Re (χ^(3)^) and imaginary Im(χ^(3)^) parts of the cubic NLO susceptibility at 1064 nm for the samples of P(VDF-TrFE) copolymer with different thickness L. Asterisk “*” marks the samples without poling.

№	Sample	L, μm	Re(*χ^(3)^*), 10^−8^ esu	Im(*χ^(3)^*), 10^−11^ esu	FOM
1	*P(VDF-TrFE)	1.0	21.1	7.5	75
2	P(VDF-TrFE)	1.2	20.2	10.3	52
3	P(VDF-TrFE)	1.3	8.6	12.3	18
4	P(VDF-TrFE)	1.5	1.3	1.8	19
5	*PVDF	40.0	0.2	−0.1	39

**Table 2:** Comparison of the obtained refractive cubic NLO response efficiency Re (*χ*^(3)^) with reference data for PVDF based materials, where RGO—reduced graphene oxide, HNT—halloysite nanotube.

Samples	λ, nm	Pulsewidth, Repetition Rate	Intensity	Re (*χ*^(3)^), esu	Ref.
PVDF/ZnO (8 wt.%)	532	7 ns, 5 Hz	1 GW/cm^2^	−1.4 × 10^−8^ 2.2 × 10^−8^	[51]
PVDF/ZnO/CuO (8/1 wt.%)					
PVDF/ZnO (1 wt.%)	532	7 ns, 5 Hz	12 GW/cm^2^	−0.4 × 10^−8^	[49]
PVDF/RGO (0.1 wt.%)	532	7 ns, 10 Hz	0.14 GW/cm^2^	3.5 × 10^−12^	[50]
Pristine PVDF	633	CW	145 W/cm^2^	−1.8 × 10^−8^ 1.2 × 10^−8^	[52]
PVDF/HNT (1%)					
PVDF	1064	42 ps, 40 Hz	1 GW/cm^2^	0.2 × 10^−8^	Present work
P(VDF-TrFE)				≥1.3 × 10^−8^	
